# Supplementary material for: Distinct population codes for attention in the absence and presence of visual stimulation
Source: Nat Commun. 2018 Oct 22;9:4382. doi: 10.1038/s41467-018-06754-5 (PMC6197235; doi:10.1038/s41467-018-06754-5)
Supplement: Supplementary file 1 — Supplementary Information [file 41467_2018_6754_MOESM1_ESM.pdf]

Supplementary materials for:

Snyder, et al.,

"Distinct population codes for attention in the absence and presence of visual stimulation"

in

Nature Communications

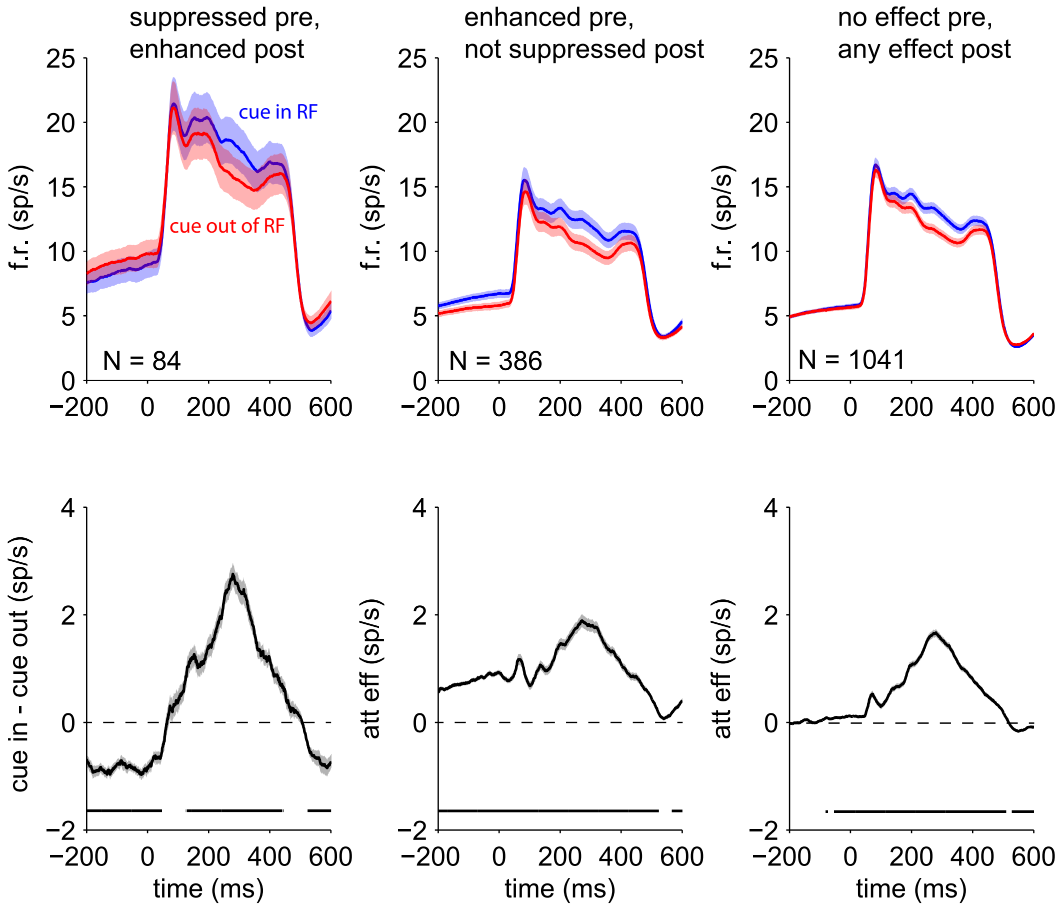

Supplementary Figure 1: peristimulus spike time histograms (PSTHs) for subpopulations of neurons selected for the pattern of their attention effects. Left: neurons that were significantly suppressed with attention pre-stimulus and significantly enhanced by attention post-stimulus. Middle: neurons that were significantly enhanced pre-stimulus and were not significantly suppressed post-stimulus. Right: neurons that were not significantly affected by attention pre-stimulus and were significantly affected (in any way) by attention post-stimulus. Notably, for all subgroups of neurons the time-course of attention effects (bottom row) follows a gradual ramp after stimulus onset, without large attention effects during the onset transient response.

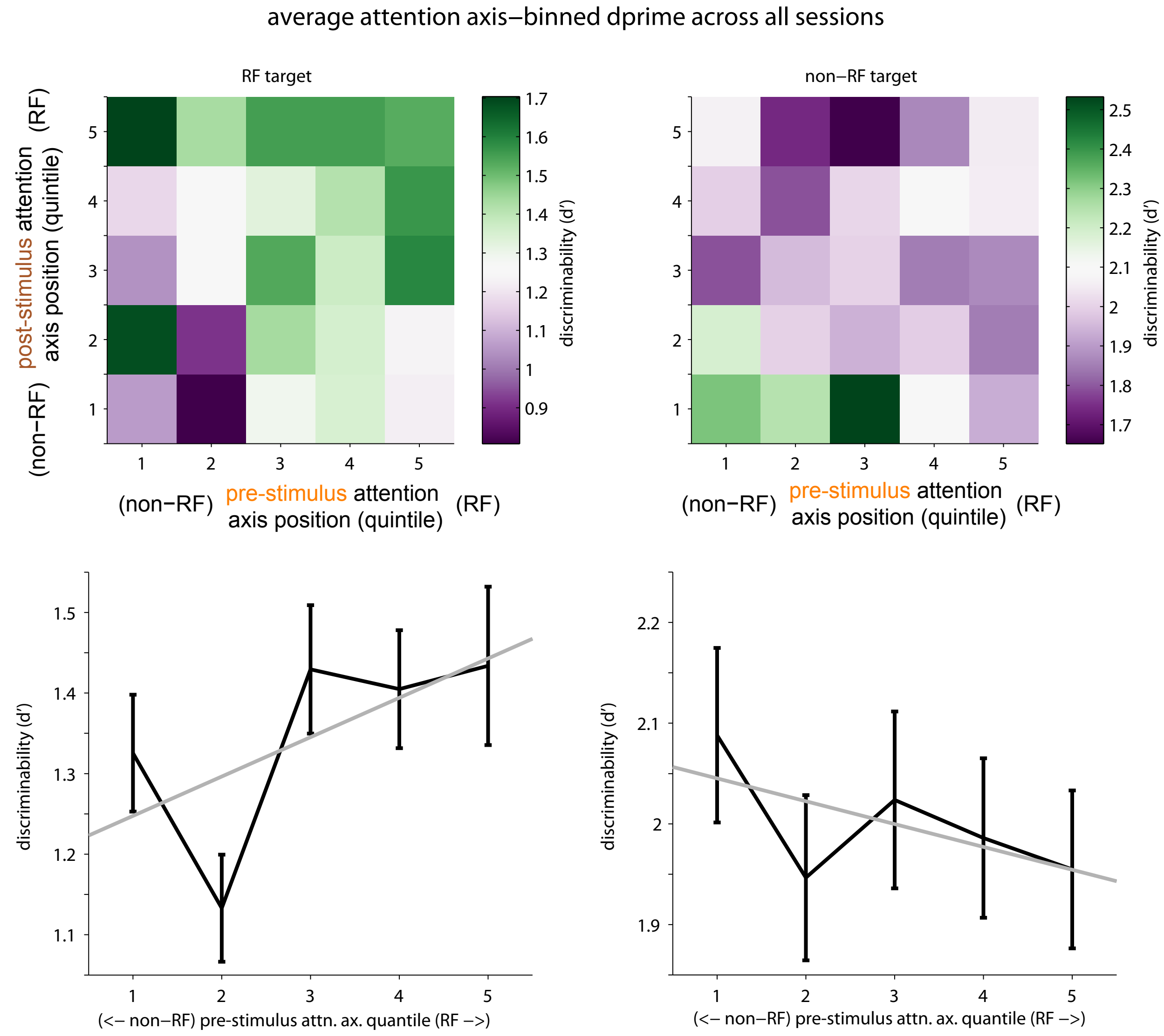

Supplementary Figure 2: Relationship between behavioral disciriminability index (d’) and attention axes projections; related to article Figure 7. Similarly to the results when hit rate was used as the metric of behavioral performance, discriminability was worst when the projection on either attention axis was consistent with attention away from the eventual target location, and improved as the projection onto either attention axis became more consistent with attention towards the eventual target location.

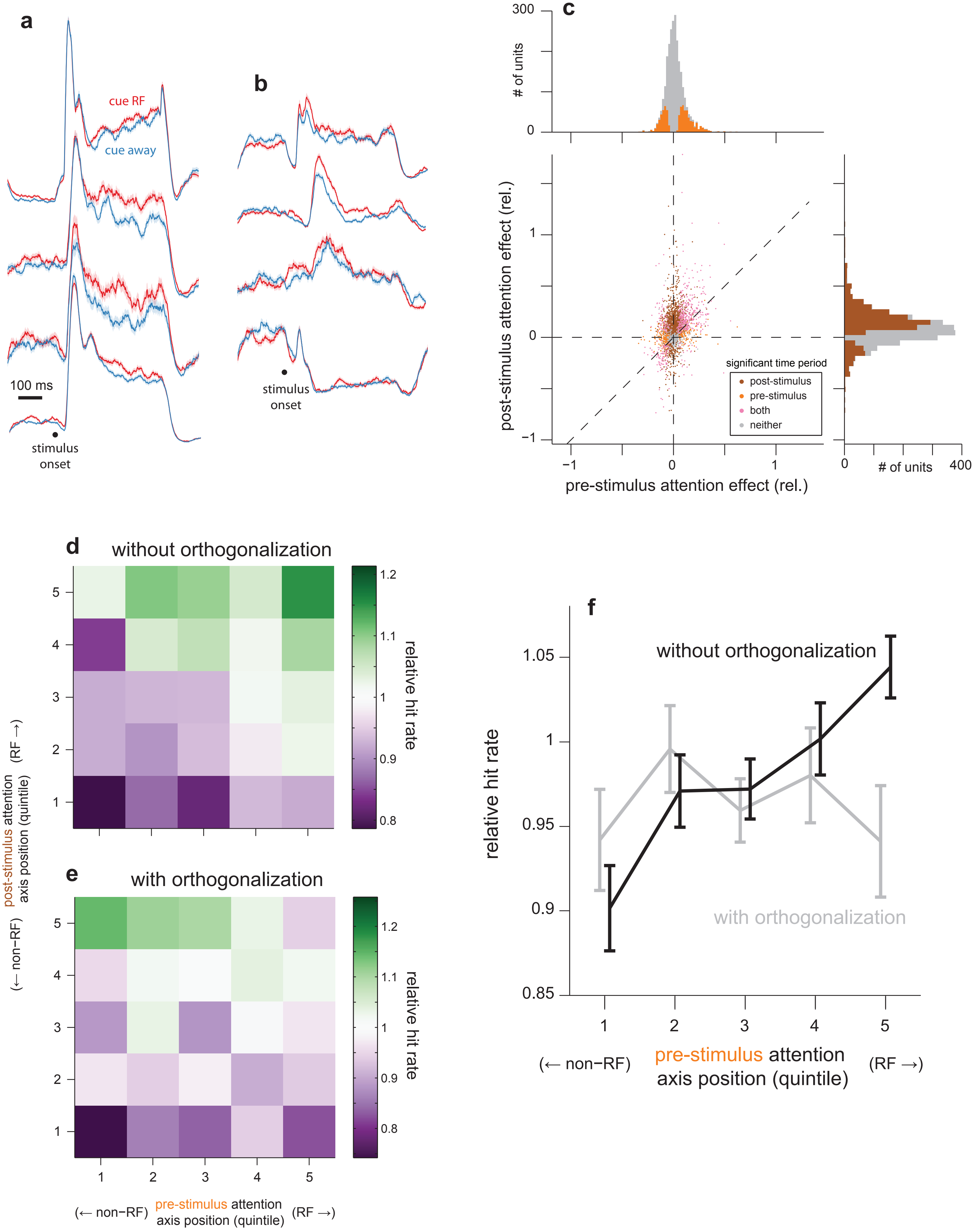

a
cue RF
cue away
100 ms
stimulus onset
b
stimulus onset
c
300
# of units
0
1
0
−1
post-stimulus attention effect (rel.)
pre-stimulus attention effect (rel.)
significant time period
post-stimulus
pre-stimulus
both
neither
400
# of units
d
without orthogonalization
relative hit rate
1.2
1.1
1
0.9
0.8
e
with orthogonalization
post-stimulus attention axis position (quintile)
(← non−RF)
(RF →)
pre-stimulus attention axis position (quintile)
f
without orthogonalization
with orthogonalization
relative hit rate
1.05
1
0.95
0.9
0.85
1
2
3
4
5

Supplementary Figure 3: illustration of analysis with simulated data. We generated surrogate data reflecting the null hypothesis that there is only one true attention axis, as for a time-invariant gain-like attention mechanism. To do this, we first found the post-stimulus and pre-stimulus attention axes as described in the manuscript (AA_{post} and AA_{pre}, respectively). We then found the rotation matrix 'R' that brings the pre-stimulus attention axis into alignment with the post-stimulus axis, that is: $AA_{post} = RAA_{pre}$. We then multiplied the vector of spike counts during the prestimulus time period of interest on each single trial by this rotation matrix, and generated new, surrogate spike trains based on these updated spike counts. We applied our analyses to these surrogate data to show that our results do not follow trivially from our approach if the null hypothesis is true.

A-B) Peristimulus spike-time histograms (PSTHs) for simulated neurons, related to Figure 3 of the main report. Vertical axes are arbitrary units of firing rate, scaled separately for each neuron. A) Most neurons in the simulated data set showed "typical" attention effects, with enhancement during the stimulus response, and slight baseline increases pre-stimulus. B) Some simulated neurons changed the direction of their attention effect between the pre-stimulus and post-stimulus time periods of interest. These examples show suppression pre-stimulus followed by enhancement. In the simulation, these "sign-flipping" neurons typically had small, noisy and disorganized visual responses, as for these examples, suggesting that finding these "sign-flipping" effects in the simulation are likely due to statistical chance. In contrast, the "sign-flipping" neurons we found in the original dataset showed more typical visual responses and clearer differences between conditions (Figure 3 in the main report).

C) Distribution of measured attention effects for the simulated data, as in Figure 4 of the main report. The distribution of pre-stimulus attention effects for the stimulated data (top) was shifted in the positive direction, compared to the distribution we observed for the real data (Figure 4 in the main report).

D) Relative hit-rate as a function of projection value on the estimate attention axes (related to Figure 7A of the main report), determined without constraining the estimated pre-stimulus attention axis to be orthogonal to the estimated post-stimulus attention axis. Behavior varies with shifts in the position along either axis.

E) Same as in (D), but with the orthogonality constraint imposed. Compared to panel (D), behavior for this analysis varies predominantly as a function of post-stimulus attention axis position only, with relatively little explanatory power added by the pre-stimulus attention axis.

F) Hit rate as a function of pre-stimulus attention axis position for simulated data (N = 47 sessions; related to Figure 7C of the main report), with and without the orthogonalization constraint imposed (gray and black, respectively). The pre-stimulus attention axis can account for substantial behavioral variation for this fixed-gain model only if the orthongalization step is not applied. Because our real experimental results showed a strong relationship with pre-stimulus attention axis position with this orthogonalization constraint (Figure 7C of the main report), we concluded that our results were inconsistent with a fixed-gain mechanism like that which was used to generate these simulated data.

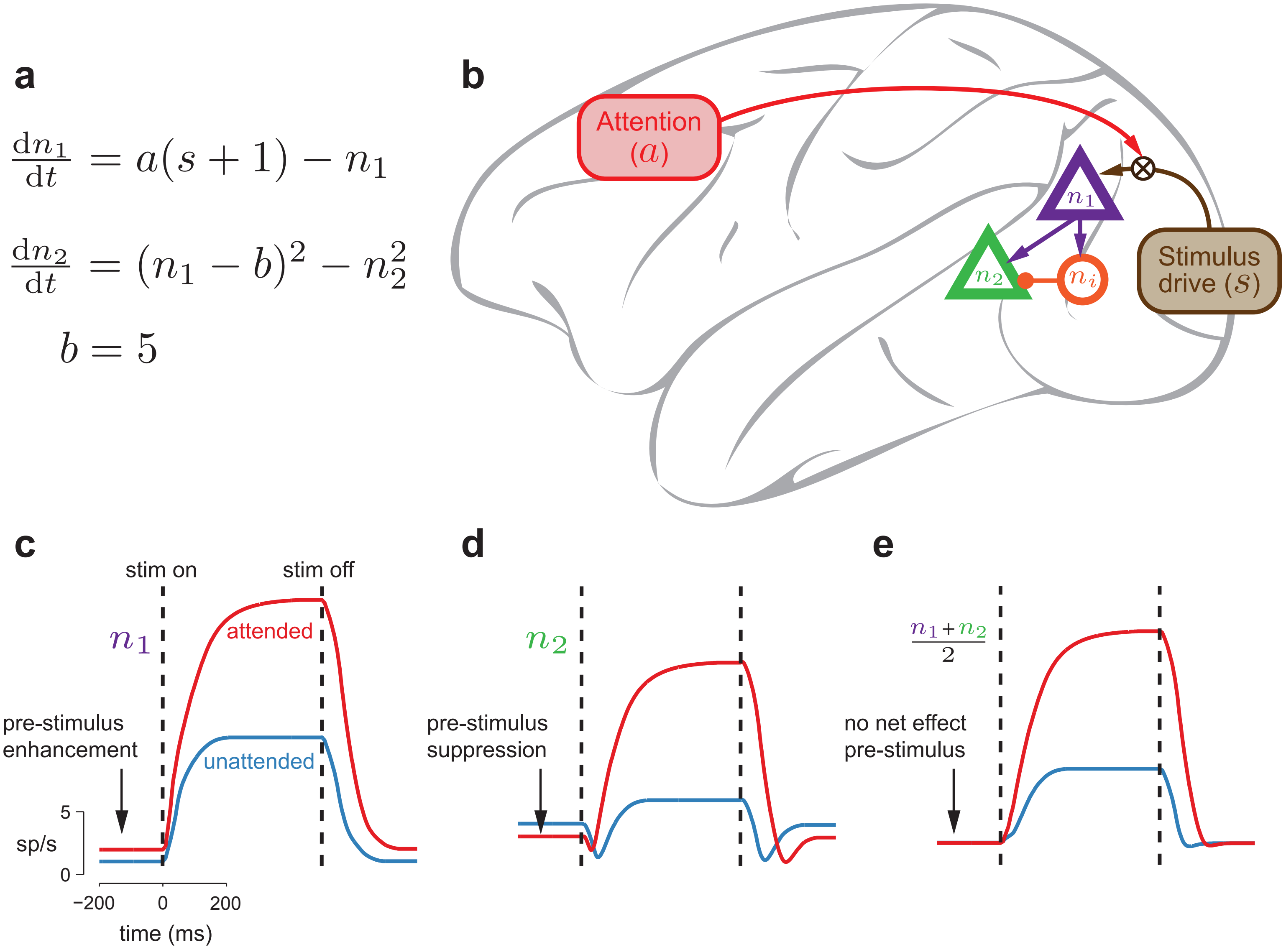

Supplementary Figure 4: Simple dynamical system model consistent with the pattern of results that we observed in the neural population data. A) system of equations governing the dynamical system. The firing rate of one neuron (n_1) depends on stimulus drive (s), which is multiplicatively scaled by an attention factor (a). The firing rate of the other neuron (n_2) depends only on the firing rate of n_1, relative to a constant value (b = 5). This latter non-linear "comparison" operation could be achieved through recruitment of local inhibitory interneurons. B) Conceptual schematic of the model. Top-down endogenous attention signals (red) interact with bottom-up stimulus drive (brown) to excite n_1 (blue), which in turn modulates the firing rate of n_2 (green), with additional involvement of local inhibitory interneurons (orange). C) Time series of firing rates for model neuron n_1 with high attention (a = 2, red) and with low attention (a = 1, blue). The neuron is facilitated by attention in both the absence (s = 0) and the presence (s = 10) of a stimulus. D) Time series of firing rates for model neuron n_2. This neuron is also facilitated by attention in the presence of a stimulus, but is suppressed by attention in the absence of a stimulus. E) Time series of the population average firing rate for the two model neurons. The population shows a typical gain-like effect in response to a stimulus, but no net effect of attention in the absence of a stimulus because of the offsetting effects of attention on the two neurons. Thus, this minimal model demonstrates that heterogenous population coupling could provide one potential mechanistic explanation for the pattern of results that we observed for the neural populations, although it does not rule out additional or alternative mechanisms.

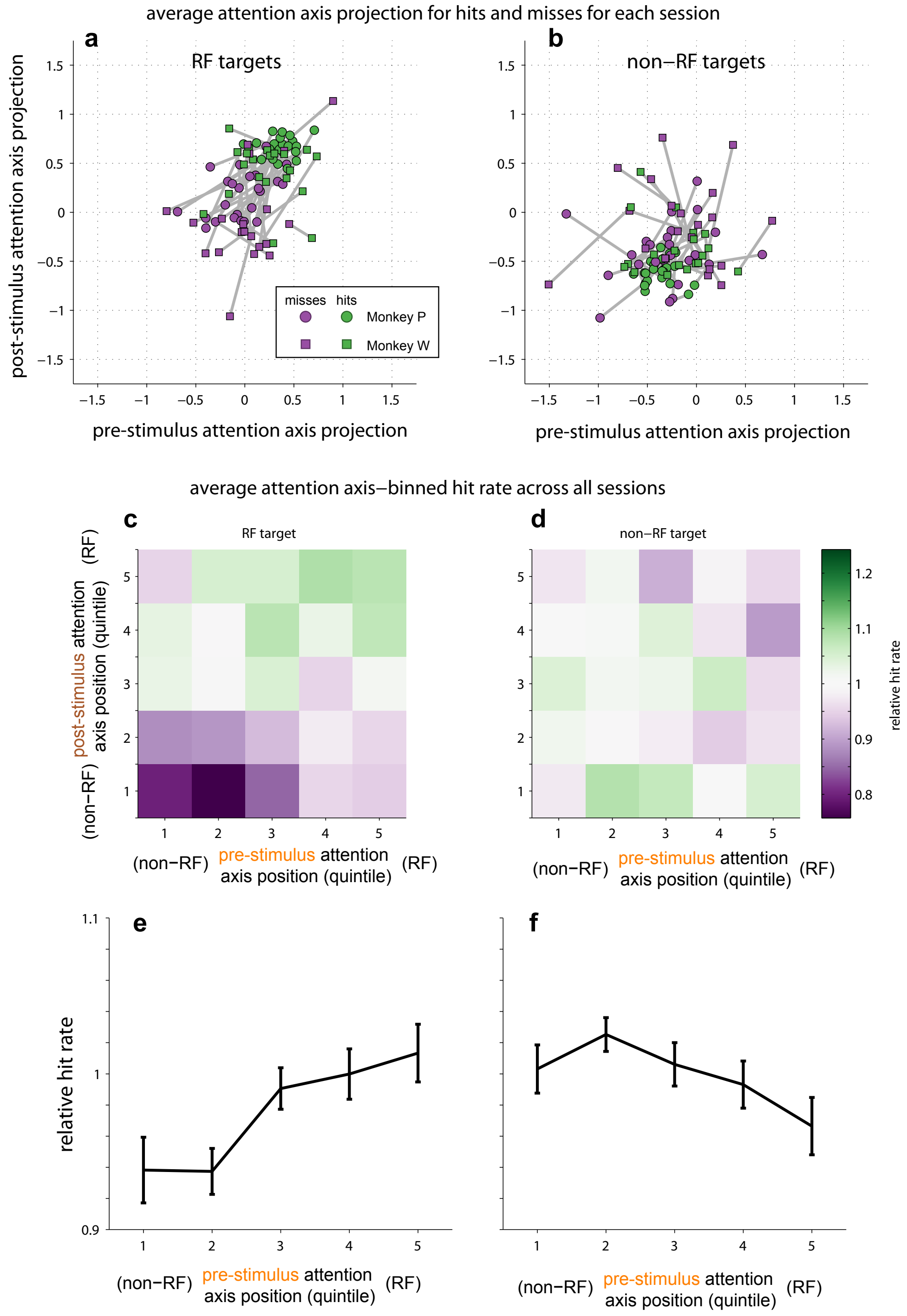

Supplementary Figure 5: main analyses performed including only well-isolated units (putative single neurons); related to article Figures 6 and 7. We quantified recording isolation by computing signal-to-noise ratios (SNRs) for each unit action potential waveform (defined as the average amplitude of the waveform divided by the standard deviation of the waveform noise), and included only units with SNR > 3. The pattern of results for this restricted analysis is similar to what we found when we did not exclude any units on the basis of SNR, as reported in the main article. (a) average attention axis projections preceding hit (green) and missed (purple) RF targets for each session. (b) average attention axis projection preceding targets out of the RF. (c) relative hit rate performance as a function of attention axis projections preceding RF targets. (d) relative hit rate performance as a function of average attention axis projections preceding targets out of the RF. (e) relative hit rate as a function of pre-stimulus attention axis projection for RF targets. (f) relative hit rate as a function of pre-stimulus attention axis projection for non-RF targets.

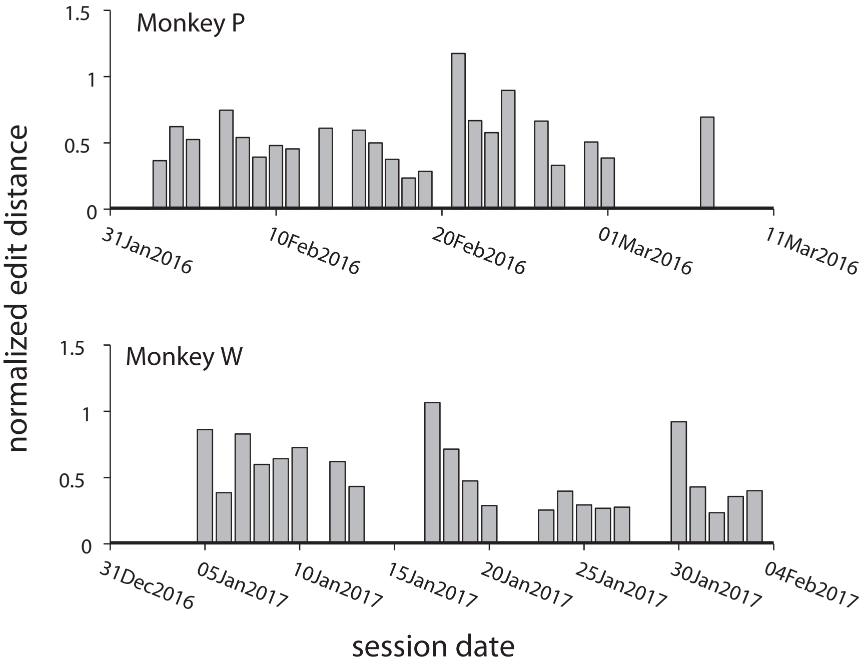

Supplementary Figure 6: the recorded sample of neurons changed substantially between sessions. We considered each session in sequence and determined the edit distance for the sample from the previous session (i.e., the number of neurons that were added or lost from the previous session). We determined that a neuron was plausibly retained across sessions if there was a neuron on the same channel with a waveform shape that was 95% correlated with a neuron on the same channel the session before. We normalized the edit distance as a proportion of the sample size of the earlier session. That is, a value of 1 means that if the sample on Monday was 100 neurons, then a total of 100 neurons were either lost or gained on Tuesday. Typically, about half the sample turned over on consecutive days. This turn-over ratio was greater if there was more time between consecutive recording sessions. Given this substantial variability across recording sessions, it is likely the consistency of results that we observed over time (e.g., article Figure 6) reflects a general property of V4 neurons, and not any one particular sample of cells.

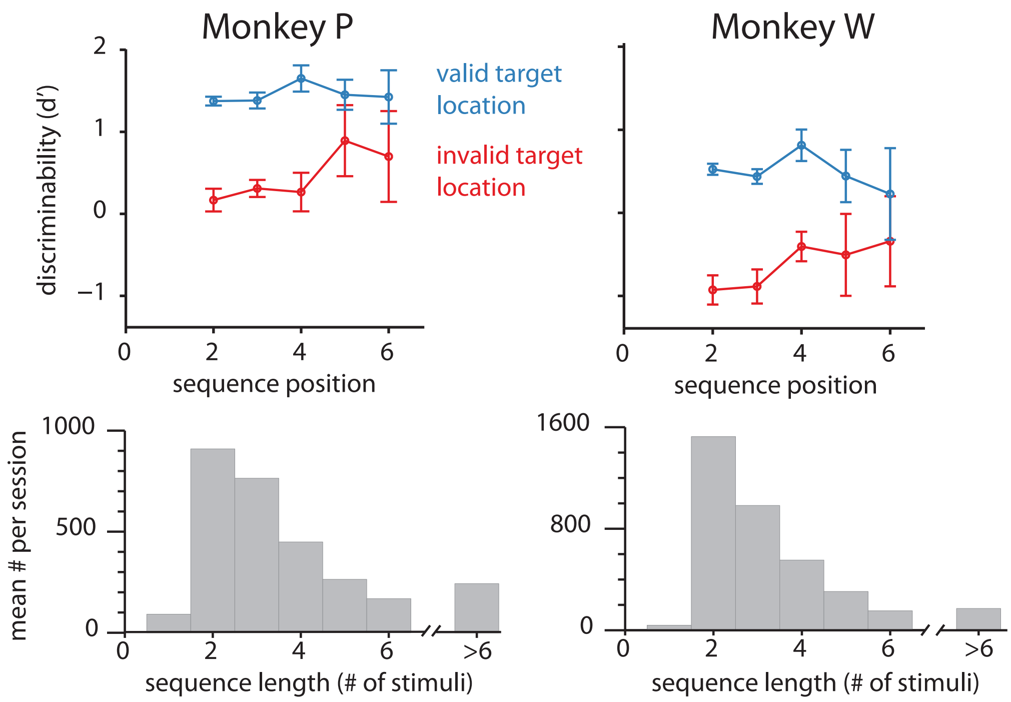

Supplementary Figure 7: Behavioral performance was consistent as a function of sequence length. Target probability followed a uniform hazard function, resulting in an exponential distribution of sequence lengths. For sequence lengths that occurred frequently enough to reliably estimate discriminability (i.e., sequences of up to 6 stimuli), behavioral performance did not vary as a function of sequence length.
